# A data-driven robust EVaR-PC with application to portfolio management

**DOI:** 10.1371/journal.pone.0287093

**Published:** 2023-06-15

**Authors:** Qingyun He, Chuanyang Hong

**Affiliations:** 1 School of Business Administration, The Southwestern University of Finance and Economics, Chengdu, China; 2 School of Computing and Artificial Intelligence, The Southwestern University of Finance and Economics, Chengdu, China; University of Pretoria, SOUTH AFRICA

## Abstract

We investigate the robust chance constrained optimization problem (RCCOP), which is a combination of the distributionally robust optimization (DRO) and the chance constraint (CC). The RCCOP plays an important role in modeling uncertain parameters within a decision-making framework. The chance constraint, which is equivalent to a constraint of Value-at-risk (VaR), is approximated by risk measures such as Entropic Value-at-risk (EVaR) or Conditional Value-at-risk (CVaR) due to its difficulty to be evaluated. An excellent approximation requires both tractability and non-conservativeness. In addition, the DRO assumes that we know partial information about the distribution of uncertain parameters instead of their known true underlying probability distribution. In this article, we develop a novel approximation EVaR- PC based on EVaR for CC. Then, we evaluate the proposed approximation EVaR- PC using a discrepancy-based ambiguity set with the wasserstein distance. From a theoretical perspective, the EVaR- PC is less conservative than EVaR and the wasserstein distance possesses many good theoretical properties; from a practical perspective, the discrepancy-based ambiguity set can make full use of the data to estimate the nominal distribution and reduce the sensitivity of decisions to priori knowledges. To show the advantages of our method, we show its application in portfolio management in detail and give the relevant experimental results.

## Introduction

In engineering and management, decision makers often need to make decisions based on uncertain parameters. Typically, for example, inventory managers need to decide on order quantities based on uncertain demands, and financial investors need to decide on investment weights based on uncertain asset returns. In general, uncertainty in these parameters may be due to limited observability of data, measurement errors, and prediction errors. In many mathematical optimization models, these uncertain parameters are assumed to be completely known nominal values according to prior knowledge or estimation from historical data. But once the real data deviates from our assumptions, it leads to poor decisions. The robust chance constrained optimization problem (RCCOP) plays an important role in modeling uncertain parameters within a decision-making framework.

The chance constraints assume that the uncertain parameters follow some unknown probability distributions and evaluate the violation probability of constraints with uncertain parameters [1–3]. The chance constraint is defined as:

$$\begin{array}{c} X_{\alpha }≔\{x\in X\;|\;\text{Pr}\{g(\xi ,x)\leq b\}\geq 1-\alpha \}, \end{array}$$ (CC)

where $x={\left(x_{1},x_{2},\ldots ,x_{p}\right)}^{⊤}\in X\subseteq R^{p}$ are the decision variables. The threshold constant *b* in the constraint are deterministic parameters. The uncertain parameters ***ξ*** = (*ξ*_1_, *ξ*_2_, …, *ξ*_*p*_)^⊤^ in the constraint are random variables with unknown probability distribution. And Pr(*A*) is the probability of an event *A* occurs and *g*(⋅) is a random function. In (CC), the constraint *g*(***ξ***, ***x***) ≤ *b* is required to be satisfied with a probability at least 1 − *α* for some 0 < *α* < 1. Taking portfolio management as an example, the vector of uncertain parameters ***ξ*** = (*ξ*_1_, *ξ*_2_, …, *ξ*_*p*_)^⊤^ represents the loss of the *p* assets, the decision variable ***x*** = (*x*_1_, *x*_2_, …, *x*_*p*_)^⊤^ is the corresponding investment weight and *b* represents the maximum loss that investors can bear. This chance constraint requires that the loss of investment *g*(***ξ***, ***x***) = ***ξ***^⊤^***x*** is less than *b* with a minimum probability of 95% if *α* = 0.05 and the violation probability Pr{*g*(***ξ***, ***x***) ≥ *b*} is required to be less than 5%.

Note that (CC) is equivalent to the value-at-risk (VaR)-based constraint:

$$\begin{array}{c} X_{\alpha }≔\{x\in X\;|\;{\text{VaR}}_{1-\alpha }\left(g\left(\xi ,x\right)\right)\leq b\}, \end{array}$$

where VaR_1−*α*_(*Y*) = inf{*s* : Pr(*Y* ≤ *s*) ≥ 1 − *α*} for any random variable *Y*. Although VaR, as an excellent risk measure, is widely used in risk control in various fields, it is neither a convex constraint nor has a closed-form. These shortcomings lead to high computational complexity to evaluate the violation probability. Many studies attempts to address this difficulty [4–6]. An extremely important method is to find convex conservative approximations to the chance constraint. Two well-known convex approximations are *conditional value-at-risk*(CVaR [7]) and the *entropic value-at-risk* (EVaR [8]), respectively. To reduce the conservatism of the approximation, [9] proposed the DC approximation, which is the difference between two convex functions. In addition to these risk measures associated with the chance constraint approximation, there are a number of risk indicators [10–13], which are beyond the scope of our paper.

The robust chance constrained optimization problem (RCCOP) evaluates the chance constraint through the paradigm of distributionally robust optimization (DRO), which assumes that the uncertain parameters belong to ambiguity sets rather than that the true probability distributions of the uncertain parameters are completely known [3]. From the perspective of DRO, decision makers need to make decisions based on the worst-case in the ambiguity set. Then, the probabilistic constraint in (CC) is turned to a robust chance constraint:

$$\begin{array}{c} \bar{X_{\alpha }}≔\{x\in X\;|\;\underset{\text{Pr}\in P}{\text{inf}}\text{Pr}\left\{g\left(\xi ,x\right)\leq b\right\}\geq 1-\alpha \}, \end{array}$$ (RCC)

where the P represents the ambiguity set containing all probability distributions with known partial information about the uncertain parameters obtained from historical data or domain-specific knowledge. The (RCC) is the feasibility set of (CC) because $x\in \bar{X_{\alpha }}$ implies $x\in X_{\alpha }$, if the true probability distribution ${\text{Pr}}^{True}\in P$.

Research on stochastic optimization assumes that the ambiguity set P only contains the true probability distribution [14–18], whereas traditional robust optimization assumes that the ambiguity set P contains all probability distributions on the support of the uncertain parameters [19–22]. However, the optimal solution may be sensitive to the pre-specific probability distribution in stochastic optimization [23–25] and may be too conservative in traditional robust optimization due to ignored other distributional information. Thus, the ambiguity set P of DRO can effectively balance advantages and disadvantages of these two optimization methods.

The ambiguity set of DRO is typically divided into two types: moment-based and discrepancy-based ambiguity sets. The moment-based ambiguity sets contain probability distributions whose moments satisfy certain properties, while discrepancy-based ambiguity sets contain probability distributions that are close to a nominal distribution in the sense of some discrepancy measure [3]. There have been many researches on moment-based ambiguity set [26]. The ambiguity set using wasserstein distance as the discrepancy measure, called Wasserstein metric of order *m*, belongs to the discrepancy-based ambiguity set. [25, 27, 28] study the DRO based on ambiguity sets of Wasserstein metrics. [29] investigates a problem of distributionally robust individual chance constraint with ambiguity sets using 1-Wasserstein metric. [25] proposes exact methods to solve the stochastic programming with a data-driven chance constraint problem. For a given historical data set, confidence sets for two types of uncertain distributions are constructed by nonparametric statistical estimation of their density functions and moments. [30] studies a data-driven distributionally robust chance-constrained problem, where the ambiguity set of the distributions is formed by the m-Wasserstein metric using arbitrary norm.

Although the ambiguity set using Wasserstein distance is a powerful modeling paradigm for characterizing uncertainty, the challenging computation arising from the non-convex feasible region due to the (CC) remains a major focus of current research. A stream of approaches to address this challenge is to identify sufficient conditions to reformulate the original problem (RCC) into a convex optimization or mixed-integer programming (MIP), which are both computationally tractable. [31] proves the convexity of the feasible region when the reference distribution of the Wasserstein ambiguity set is Gaussian or log-concave. They derive a convex and conic representation and propose a block coordinate ascent algorithm, respectively, to effectively solve the chance constraint problems. [32] derives tractable mixed-integer linear or conic programming reformulations for several variants of distributionally (RCC) with Wasserstein ambiguity set under two types of uncertainties (i.e., uncertain probabilities vs. continuum of realizations). [33] also provide a mixed-integer conic program reformulation for chance constraint with right-hand side uncertainty. Meanwhile, as mentioned before, replacing chance constraint with its convex conservative approximations is also an important approach to transform the (CC) into convex optimization. The significance of our proposed EVaR- PC lies in improving the conservatism of the widely used convex conservative approximation EVaR and demonstrating its computational tractability in portfolio management.

The contributions of this paper contain the following.

1. A tighter upper bound EVaR- PC based on EVaR for violation probability is developed, which takes existing approximation approaches as a special case.
2. We show how to evaluate the proposed approximation EVaR- PC based on the m-Wasserstein metric using arbitrary norm.
3. This article explains how to apply our proposed model of RCCOP to portfolio management, and illustrates the advantages of our method with experimental results.

### Outline

This paper is organized as follows. In §Methods, we propose the EVaR- PC approximation and introduce the m-Wasserstein metric-based ambiguity sets for DRO. An application in portfolio management is shown in §Portfolio management. In §Conclusions, we give a summary of this article and potential research content in the future.

## Methods

In this section, we propose a general family of upper-bounds which enjoy nice properties of the DC approximation but are more flexible to introduce more variants, and EVaR- PC approximation is a member of this family. In order to evaluate these conservative approximations in the context of DRO, we then introduce the definition of the discrepancy-based ambiguity sets using m-Wasserstein metric and related reformulation for final the optimization problem.

### A tighter upper bound for violation probability

As described in the previous section, the (CC) is equivalent to the VaR-based constraint. The CVaR and EVaR are two conservative approximations to VaR. Specifically, the approximations for VaR-based constraint are

$$\begin{array}{c} X_{\alpha }≔\{x\in X\;|\;{\text{CVaR}}_{1-\alpha }\left(g\left(\xi ,x\right)\right)\leq b\} \end{array}$$

and

$$\begin{array}{c} X_{\alpha }≔\{x\in X\;|\;{\text{EVaR}}_{1-\alpha }\left(g\left(\xi ,x\right)\right)\leq b\}, \end{array}$$

where ${\text{CVaR}}_{1-\alpha }\left(Y\right)={\text{inf}}_{s>0}\{s+\frac{1}{\alpha }E{\left[Y-s\right]}^{+}\}$ and ${\text{EVaR}}_{1-\alpha }\left(Y\right)={\text{inf}}_{s>0}\{\frac{1}{s}\text{ln}(\alpha^{-1}E[e^{sY}])\}$ for any random variable *Y*.

From the perspective of violation probability, the $\text{Pr}\left\{g\left(\xi ,x\right)>b\right\}=E[1_{\left(b,\infty \right)}(g(\xi ,x))]$, where $1_{A}\left(s\right)$ is an indicator function such that it equals to 1 if *s* ∈ *A* and 0 otherwise. If $\Phi \left(g\left(\xi ,x\right)-b\right)\geq 1_{\left(b,\infty \right)}(g(\xi ,x))$, the E[Φ(g(ξ,x)-b)] can be an upper bound for violation probability Pr{*g*(***ξ***, ***x***) > *b*}. Then, the approximated robust chance constraint

$$\begin{array}{c} \bar{\bar{X_{\alpha }}}≔\{x\in X\;|\;\underset{\text{Pr}\in P}{\text{sup}}\;E[\Phi (g(\xi ,x)-b)]\leq \alpha \} \end{array}$$ (ARCC)

guarantees the feasible solution of (CC). For CVaR and EVaR,

$$\begin{array}{c} \Phi_{\text{CVaR}}\left(g\left(\xi ,x\right)-b,t\right)=\frac{1}{t}{\left(t+g(\xi ,x)-b\right)}^{+} \end{array}$$ (1)

and

$$\begin{array}{c} \Phi_{\text{EVaR}}\left(g\left(\xi ,x\right)-b,t\right)=e^{[g(\xi ,x)-b]/t}, \end{array}$$ (2)

where *t* > 0 and *s*^+^ = max(*s*, 0) for any s∈R.

As discussed in §Introduction, many existing literature have proposed good upper-bounds (or conservative approximations) for the violation probability under the (ARCC) setting, such as CVaR approximation (1) by [8], and EVaR approximation (2) by [8], among others. A distinct approach proposed by [9] uses a difference between two convex functions to define the DC upper-bound:

$$\begin{array}{c} \Phi_{\text{DC}}\left(g\left(\xi ,x\right)-b,t\right)=\frac{1}{t}[{\left(t+g(\xi ,x)-b\right)}^{+}-{\left(g(\xi ,x)-b\right)}^{+}]. \end{array}$$ (3)

Although the resulting approximation is not convex, it is much tighter than other convex approximations. The original motivation of [9] is first to “shift Φ_CVaR_(*g*(***ξ***, ***x***) − *b*, *t*) to the right side by a distance of *t*”. We observe that the resulting function is equivalent to “the positive part of shifting Φ_CVaR_(*g*(***ξ***, ***x***) − *b*, *t*) down by a distance of 1”. That is $\frac{1}{t}{\left(g(\xi ,x)-b\right)}^{+}={\left[\Phi_{\text{CVaR}}\left(g\left(\xi ,x\right)-b,t\right)-1\right]}^{+}$. Inspired by such an approach, we propose a general family of upper-bounds which enjoy nice properties of the DC approximation but are more flexible to introduce more variants.

Without losing generality, we study the mathematical properties of these upper-bounds by assuming *b* = 0 and linear relationship *g*(***ξ***, ***x***) = ***ξ***^⊤^***x***, which is very suitable for portfolio management. Let Φ_0_(***ξ***^⊤^***x***, *t*) be any upper-bound of $1_{\left(0,\infty \right)}\left(\xi^{⊤}x\right)$ where *t* > 0 such that $\Phi_{0}\left(\xi^{⊤}x,t\right)\geq 1_{\left(0,\infty \right)}\left(\xi^{⊤}x\right)$ for any $\xi^{⊤}x\in R$. We then define the following family of approximations for the indicator function by taking the difference between the original upper-bound and its vertical shift as

$$\begin{array}{c} C[\Phi_{0}\left(\xi^{⊤}x,t\right)]=\Phi_{0}\left(\xi^{⊤}x,t\right)-{\left[\Phi_{0}\left(\xi^{⊤}x,t\right)-1\right]}^{+}. \end{array}$$ (4)

Since the upper-bounds in our proposed family always take value 1 for ***ξ***^⊤^***x*** ≥ 0, we call (4) a positive-capped (PC) approximation. For any *t* > 0, $C[\Phi_{0}\left(\xi^{⊤}x,t\right)]$ is a conservative approximation of Pr(***ξ***^⊤^***x*** > *b*).

Hence, we can let $\Phi_{\text{PC}}\left(\xi^{⊤}x,t\right)={\text{inf}}_{t>0}\{C[\Phi_{0}\left(\xi^{⊤}x,t\right)]\}$, which is the best approximation among all.

**Lemma 1**. *For any* Φ_0_(***ξ***^⊤^***x***, *t*) *that is monotonic nondecreasing in t* > 0, $C[\Phi_{0}\left(\xi^{⊤}x,t\right)]$ *is nondecreasing in t for any t* > 0.

Proof of Lemma 1. For any 0 < *t*_2_ < *t*_1_ and any $\xi^{⊤}x\in R$, it holds that Φ_0_(***ξ***^⊤^***x***, *t*_2_) < Φ_0_(***ξ***^⊤^***x***, *t*_1_), and

$$\begin{array}{c} C[\Phi_{0}\left(\xi^{⊤}x,t_{1}\right)]-C[\Phi_{0}\left(\xi^{⊤}x,t_{2}\right)]\\ =\Phi_{0}\left(\xi^{⊤}x,t_{1}\right)-\Phi_{0}\left(\xi^{⊤}x,t_{2}\right)+{\left[\Phi_{0}\left(\xi^{⊤}x,t_{2}\right)-1\right]}^{+}-{\left[\Phi_{0}\left(\xi^{⊤}x,t_{1}\right)-1\right]}^{+}\\ =\{\begin{array}{cc} \Phi_{0}\left(\xi^{⊤}x,t_{1}\right)-\Phi_{0}\left(\xi^{⊤}x,t_{2}\right) & \;\text{if}\;\Phi_{0}\left(\xi^{⊤}x,t_{2}\right)<\Phi_{0}\left(\xi^{⊤}x,t_{1}\right)\leq 1,\\ 1-\Phi_{0}\left(\xi^{⊤}x,t_{2}\right) & \;\text{if}\;\Phi_{0}\left(\xi^{⊤}x,t_{2}\right)\leq 1<\Phi_{0}\left(\xi^{⊤}x,t_{1}\right),\\ 0 & \;\text{if}\;1<\Phi_{0}\left(\xi^{⊤}x,t_{2}\right)<\Phi_{0}\left(\xi^{⊤}x,t_{1}\right). \end{array} \end{array}$$

which is always nonnegative. Therefore, $C[\Phi_{0}\left(\xi^{⊤}x,t\right)]$ is nondecreasing in t for any *t* > 0.

It can be seen that the DC approximation (3) belongs to this general family by setting Φ_0_(***ξ***^⊤^***x***, *t*) to be Φ_CVaR_(***ξ***^⊤^***x***, *t*). That is $\Phi_{\text{DC}}\left(\xi^{⊤}x,t\right)=\Phi_{\text{CVaR-PC}}\left(\xi^{⊤}x,t\right)=C[\Phi_{\text{CVaR}}\left(\xi^{⊤}x,t\right)]$. Similarly, we can use the EVaR approximation (2) in (4) to obtain the EVaR based PC approximation as

$$\begin{array}{c} \Phi_{\text{EVaR-PC}}\left(\xi^{⊤}x,t\right)=C[\Phi_{\text{EVaR}}\left(\xi^{⊤}x,t\right)]=e^{\xi^{⊤}x/t}-{\left[e^{\xi^{⊤}x/t}-1\right]}^{+}. \end{array}$$ (EVaR-PC)

**Fig 1:**
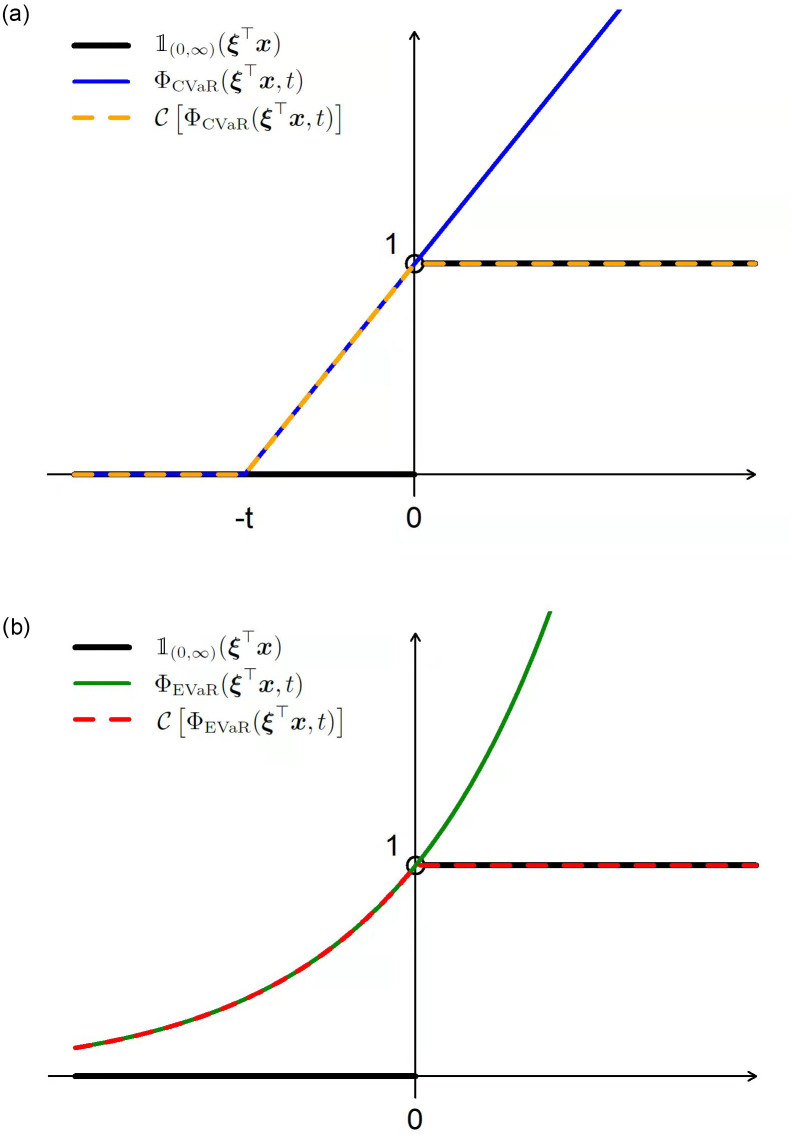
Comparisons of different approximations for the indicator function. Left: This plot compares the convex approximation CVaR for the indicator function to its corresponding PC approximation that is equivalent to the DC approximation proposed by [9]. Right: This plot compares the convex approximation EVaR for the indicator function to its corresponding PC approximation.

Fig 1 provides a comparison with different approximations for the indicator function. Although not being convex anymore, the PC family generally provides a much tighter upper-bound than many conventional convex approximations, such as CVaR (1) and EVaR (2). We also want to make a remark that while both being members of the PC family, the EVaR-PC approximation contains only one non-differentiable point at ***ξ***^⊤^***x*** = 0 whereas the DC approximation, equivalent to CVaR-PC approximation, has two non-differentiable points at ***ξ***^⊤^***x*** = 0 and ***ξ***^⊤^***x*** = −*t*.

**Theorem 1**. *Let* E[Φ(g(ξ,x)-b)] *in* (ARCC) *be* ${\text{inf}}_{t>0}\{E(\Phi_{\text{EVaR-PC}}\left(g\left(\xi ,x\right)-b,t\right))\}$. *Then* (ARCC) *converges to* (RCC).

Proof of Theorem 1. When *g*(***ξ***, ***x***) − *b* ≤ 0, the Φ_EVaR_(*g*(***ξ***, ***x***) − *b*, *t*) is monotonic nondecreasing in *t* > 0. The $C[\Phi_{\text{EVaR}}\left(g\left(\xi ,x\right)-b,t\right)]$ is nondecreasing in *t* for any *t* > 0. Hence,

$$\begin{array}{cc} \underset{t>0}{\text{inf}}\{E(\Phi_{\text{EVaR-PC}}\left(g\left(\xi ,x\right)-b,t\right))\} & =\underset{t>0}{\text{inf}}\{E(C[\Phi_{\text{EVaR}}\left(g\left(\xi ,x\right)-b,t\right)])\}\\ & =\underset{t>0}{\text{inf}}\{E(e^{(g(\xi ,x)-b)/t}-[e^{(g(\xi ,x)-b)/t}-1]{(}^{+})\}\\ & =\underset{t↘0}{\text{lim}}\{E(e^{(g(\xi ,x)-b)/t}\cdot I_{\left(-\infty ,b\right]}\left(g\left(\xi ,x\right)\right)+I_{\left(b,+\infty \right)}\left(g\left(\xi ,x\right)\right))\}\\ & =E(I_{\left(b,+\infty \right)}\left(g\left(\xi ,x\right)\right))\\ & =\text{Pr}\{g(\xi ,x)>b\}=1-\text{Pr}\{g(\xi ,x)\leq b\}, \end{array}$$

which completes the proof.

Theorem 1 provides a connection between the proposed novel upper-bounds in the PC family and the original chance constraint. This conclusion is also applicable to the special case of *b* = 0 and linear relationship *g*(***ξ***, ***x***) = ***ξ***^⊤^***x***.

### The m-Wasserstein metric

The Wasserstein distance is a function that can measure the distance between probability distributions on a given metric space and is also called the “earth mover’s distance”. If each distribution is viewed as a unit amount of earth piled on the given metric space and we need to turn one pile into the other, Wasserstein distance is assumed to be the minimum amount of earth that needs to be moved times the mean distance it has to be moved.

**Definition 1**. *The f*_*Y*_ *is the probability density function (p.d.f.) for continuous random variable Y* ∈ Ξ. *Then, for m* ≥ 1, *the m-Wasserstein distance* $W_{m}\left(f_{Y^{1}},f_{Y^{2}}\right)$ *between* $f_{Y^{1}},f_{Y^{2}}\in P\left(\Xi \right)$ *is defined by*

$$\begin{array}{c} W_{m}\left(f_{Y^{1}},f_{Y^{2}}\right)≔{\left(\underset{\gamma \in \Gamma (f_{Y^{1}},f_{Y^{2}})}{\text{inf}}\int_{\Xi \times \Xi }d{\left(Y^{1},Y^{2}\right)}^{m}d\gamma \left(Y^{1},Y^{2}\right)\right)}^{\frac{1}{m}}, \end{array}$$ (5)

*where* $\Gamma \left(f_{Y^{1}},f_{Y^{2}}\right)$ *denotes the collection of all measures on* Ξ × Ξ *with marginals* $f_{Y^{1}}$ *and* $f_{Y^{2}}$ *on the first and second factors respectively*.

**Definition 2**. *The p*_*Y*_(*y*) = Pr(*Y* = *y*) *is the probability mass function (p.m.f.) for discrete random variable Y on finite domain* Ξ. *For m* ≥ 1, *the m-Wasserstein distance in* (5) *becomes*

$$\begin{array}{c} W_{m}\left(p_{Y^{1}},p_{Y^{2}}\right)≔{\left(\underset{\gamma (Y^{1},Y^{2})\geq 0}{\text{inf}}\sum_{Y^{1}\in \Xi }\sum_{Y^{2}\in \Xi }d{\left(Y^{1},Y^{2}\right)}^{m}\gamma \left(Y^{1},Y^{2}\right)\right)}^{\frac{1}{m}}, \end{array}$$

*where* $\sum_{Y^{1}\in \Xi }\gamma (Y^{1},Y^{2})=p_{Y^{2}}$ *for* ∀*Y*^2^ ∈ Ξ *and* $\sum_{Y^{2}\in \Xi }\gamma (Y^{1},Y^{2})=p_{Y^{1}}$ *for* ∀*Y*^1^ ∈ Ξ.

Next, we present methods for solving optimization problems with discrepancy-based ambiguity sets using m-Wasserstein metric. The decision making problem can be formulated as follows:

$$\begin{array}{c} \underset{x\in X}{\text{inf}}\;\underset{f_{\xi }\in P}{\text{sup}}E_{f_{\xi }}\left[\Psi \left(\xi ,x\right)\right]\;\text{and}\;\;P≔\{f\in P\left(\Xi \right)\;|\;W_{m}\left(f,f_{0}\right)\leq \theta \}, \end{array}$$ (6)

where *θ* > 0 is a chosen radius and $\xi \in \Xi \subseteq R^{p}$. And the *f*_0_ is a nominal distribution, such as an empirical distribution, that estimated by historical data. To solve the problem (6), [34] proves the strong duality result using a novel, elementary, constructive approach. For the completeness of our article, we show one of their dual results for finite domain, which is also closely related to our research.

**Example 1**. *When* Ξ *is finite, that is* Ξ = {***ξ***_1_, ***ξ***_2_, …, ***ξ***_*N*_}, *the nominal distribution p*_0_ *is given by p*_0_(***ξ***_*j*_) *for* *j* = 1, 2, …, *N*. *Then, the problem* (6) *becomes*:

$$\begin{array}{c} \underset{x\in X}{\text{min}}\;\underset{p\left(\xi_{1}\right),\ldots ,p\left(\xi_{N}\right)}{\text{max}}\{\sum_{j=1}^{N}p\left(\xi_{j}\right)\Psi \left(\xi_{j},x\right)\;|\;W_{m}\left(p,p_{0}\right)\leq \theta \}, \end{array}$$ (7)

*which has a strong dual*

$$\begin{array}{c} \underset{x\in X,\lambda \geq 0,s≔{\left(s_{1},\ldots ,s_{N}\right)}^{⊤}}{\text{min}}\{\lambda \theta^{m}+\sum_{j=1}^{N}p_{0}\left(\xi_{j}\right)s_{j}\;|\;s_{j}\geq \Psi \left(\xi_{k},x\right)-\lambda d{\left(\xi_{j},\xi_{k}\right)}^{m},\;\forall j,k=1,\ldots ,N\}. \end{array}$$ (8)

*For any given p*_0_(***ξ***_*j*_) *for* *j* = 1, 2, …, *N and* ***x***, *the objective and worst-case distribution of the m-Wasserstein metric-based ambiguity set of the original problem* (7) *can be obtained by*

$$\begin{array}{cc} \underset{p\left(\xi_{1}\right),\ldots ,p\left(\xi_{N}\right),\gamma_{j,k}>0\;\forall j,k=1,\ldots ,N}{\text{max}}\{\sum_{j=1}^{N}p\left(\xi_{j}\right)\Psi \left(\xi_{j},x\right)\;|\;\sum_{j=1}^{N}\sum_{k=1}^{N}d{\left(\xi_{j},\xi_{k}\right)}^{m}\gamma_{j,k}\leq \theta^{m}, & \\ \sum_{j=1}^{N}\gamma_{j,k}=p_{0}\left(\xi_{k}\right)\forall k,\sum_{k=1}^{N}\gamma_{j,k}=p\left(\xi_{j}\right)\forall j\}, \end{array}$$ (9)

*where* *γ*_*j*,*k*_ = *γ*(***ξ***_*j*_, ***ξ***_*k*_).

## Portfolio management

In this section, we first introduce the experimental data and the portfolio investment management strategy, and then demonstrate and discuss the results of the data experiment.

### Experimental data and algorithm

Our portfolio management strategy is based on financial assets, and the experimental data are from the financial client Wind. We select 5 globally important market indexes as investment objects. Their codes in the financial client Wind are as follows: IBOVESPA.GI, IXIC.GI, FTSE.GI, AS51.GI and 000001.SH. The experimental data are the weekly returns of these market indexes from October 2017 to October 2022, and our strategy requires weekly adjustment of investment weights based on changes in these weekly data.

In our experiment, ***ξ*** represents loss or negative return, the decision variable ***x*** represents investment weight and then *g*(***ξ***, ***x***) = ***ξ***^⊤^***x***. We use historical data from the past year to estimate ***ξ***, so the elements in the set Ξ = {***ξ***_1_, ***ξ***_2_, …, ***ξ***_*N*_} are the losses over the past *N* = 52 weeks. The empirical distribution $p_{0}\left(\xi_{j}\right)=\frac{1}{N}$ for *j* = 1, 2, …, *N* is used as the nominal distribution for m-Wasserstein metric. The problem (7) is our primary original experimental model. In addition, a supplementary constraint **1**^⊤^***x*** = 1 for ***x*** ≥ **0** is added to indicate that short selling is not allowed. For the hyperparameters, let *m* = 1 to simplify the calculation, *b* = 0 and *θ* selected from set {0.1, 0.5, *θ*_*N*_(*β*)}, where *θ*_*N*_(*β*) is the minimum radius of Wasserstein ball that contains the true distribution with confidence 1 − *β* for some prescribed *β* ∈ (0, 1) based on a finite sample (i.e., N = 52) guarantee. According to the results from [35], we have

$$\begin{array}{cc} \theta_{N}\left(\beta \right) & =\{\begin{array}{cc} {\left(\frac{{\text{log}}_{\;}\left(c_{1}\beta^{-1}\right)}{c_{2}N}\right)}^{\frac{1}{\text{max}\{M,2\}}} & \;\text{if}\;N\geq \frac{{\text{log}}_{\;}\left(c_{1}\beta^{-1}\right)}{c_{2}},\\ {\left(\frac{{\text{log}}_{\;}\left(c_{1}\beta^{-1}\right)}{c_{2}N}\right)}^{\frac{1}{a}} & \;\text{if}\;N<\frac{{\text{log}}_{\;}\left(c_{1}\beta^{-1}\right)}{c_{2}}, \end{array} \end{array}$$

for all *N* ≥ 1, *M* ≠ 2 and *θ* > 0. The *c*_1_ and *c*_2_ are positive constants, which are set to 0.1 and 2 respectively. Let *M* < 2 and *β* = 0.05. And then, it holds that the minimum radius *θ*_*N*_(*β*) ≈ 0.0816.

The main experimental algorithm steps are as follows:

1. Let Ψ(***ξ***, ***x***) = Φ_EVaR_(***ξ***^⊤^***x***, *t*) for (7). Solve the strong dual problem (8) to get its optimal solution x*.
2. For given empirical distribution $p_{0}\left(\xi_{j}\right)=\frac{1}{N}$ for *j* = 1, 2, …, *N* and x*, we solve problem (9) to obtain the worst-case distribution p*(***ξ***_*j*_) for *j* = 1, 2, …, *N* in ambiguity set.
3. Based on the worst-case distribution p*(***ξ***_*j*_) and $\Phi_{\text{EVaR-PC}}\left(\xi_{j}^{⊤}x^{*},t\right)$, we can have ${\text{sup}}_{p_{\xi }\in P}E_{p_{\xi }}\Phi_{\text{EVaR-PC}}\left(\xi^{⊤}x^{*},t\right)=\sum_{j=1}^{N}p^{*}\left(\xi_{j}\right)\Phi_{\text{EVaR-PC}}\left(\xi_{j}^{⊤}x^{*},t\right)$.
4. Let *α* = 0.05, 0.1 and 0.2. If the $\sum_{j=1}^{N}p^{*}\left(\xi_{j}\right)\Phi_{\text{EVaR-PC}}\left(\xi_{j}^{⊤}x^{*},t\right)\leq 0.05$, investment weights $\hat{x}=2x^{*}$; If the $\sum_{j=1}^{N}p^{*}\left(\xi_{j}\right)\Phi_{\text{EVaR-PC}}\left(\xi_{j}^{⊤}x^{*},t\right)\geq 0.2$, investment weights $\hat{x}=0$; otherwise, investment weights $\hat{x}=x^{*}$.
5. Calculate out-of-sample return performance for the weekly strategy by the investment weights $\hat{x}$.

It is worth noting that the optimal *t* for ${\text{inf}}_{t>0}\{E(\Phi_{\text{EVaR}}\left(\xi^{⊤}x,t\right))\}$ can be obtained by searching method [36]. To better reflect the experimental performance of our proposed method, we use two benchmarks as comparison. We show in detail how these two benchmarks are calculated:

1. **MV**: min_***x***_***x***^⊤^∑***x*** and $\sum_{i}^{p}x_{i}=1$ for *x*_*i*_ ≥ 0, *i* = 1, …, *p*. The ∑ represents the covariance matrix of the returns on *p* assets.
2. **Mean**: Let $x_{i}=\frac{1}{p}$ for *i* = 1, …, *p*. This method can reflect the average return of these investment objects.

### Experimental results and discussion

As mentioned above, an excellent approximation requires both tractability and non-conservativeness. Although, CVaR is the “tightest” approximation, it has no explicit closed-form. EVaR is very solvable due to its convexity, but it’s a very conservative approximation. Therefore, we propose EVaR- PC approximation based on EVaR, which can effectively reduce the conservatism of EVaR. The EVaR- PC approximation is a tighter upper bound for violation probability, which means it is a risk measure. In our model setting, the value of EVaR- PC represents the upper bound on the probability that an investment strategy may lose money. For example, if ${\text{inf}}_{t>0}\{E[\Phi_{\text{EVaR-PC}}\left(\xi^{⊤}x,t\right)]\}=0.05$ for *b* = 0, it means that the current investment strategy may suffer losses with a probability of less than 5%.

The main purpose of our experiment is to examine two questions: (1) Will the EVaR- PC-based strategy lead to an improvement in the performance of the EVaR-based strategy? (2) What are the effects of different risk thresholds on the experimental results? To answer these two questions, we present the net asset value curves of different strategies and calculate the corresponding measures that are widely used in the financial field. The whole experiment tested the performance of weekly investment 204 times, so the net asset value is defined as $\prod_{t=1}^{T}\left(1-\xi_{t}^{⊤}x\right)$ for *T* = 1, …, 204, where −***ξ***_*t*_ is the return of the asset at week *t*. The Maximum Drawdown is one of the important financial indicators of the maximum loss that investors may suffer over the investment horizon.

**Fig 2:**
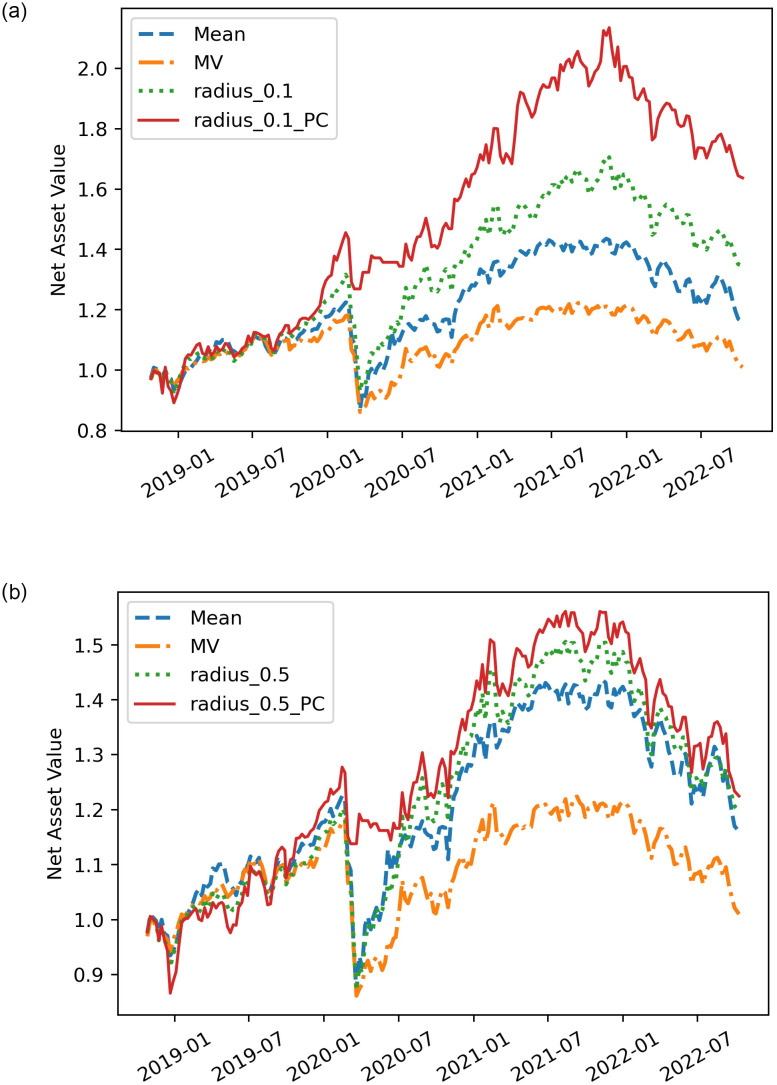
Comparisons of different net asset value curves for different approximations. Left: *θ* = 0.1. The method radius_0.1 is a EVaR-based strategy calculated by weights x*, while radius_0.1_PC is calculated by its corresponding weights $\hat{x}$. Right: *θ* = 0.5. The method radius_0.5 is a EVaR-based strategy calculated by weights x*, while radius_0.5_PC is calculated by its corresponding weights $\hat{x}$.

The results in Fig 2 and Table 1 can answer the first question: The EVaR- PC-based strategy lead to a significant improvement in the performance of the EVaR-based strategy.

**Table 1:** The performance of different methods and benchmarks (Annualization).

	*Returns*	*Std*	*Sharpe*_*ratio*	*MaximumDrawdown*
Mean	5.21%	0.17	0.31	28.95%
MV	1.27%	0.14	0.09	27.13%
radius_0.1	9.15%	0.18	0.52	29.42%
radius_0.1_PC	14.17%	0.18	0.79	23.31%
radius_0.5	5.97%	0.16	0.36	27.51%
radius_0.5_PC	6.48%	0.16	0.40	21.54%
radius_min	9.21%	0.18	0.52	29.58%
radius_min_PC	9.23%	0.18	0.52	20.97%

It can be seen that radius_0.1_PC’s and radius_0.5_PC’s net asset value curves are always above radius_0.1’s and radius_0.5’s respectively throughout the experimental period in Fig 2. Higher net asset value means better performance in terms of return on assets. In addition, in the Table 1, radius_0.1 and radius_0.5 have annualized returns of 9.15% and 5.97%, while radius_0.1_PC and radius_0.5_PC can achieve annualized returns of 14.17% and 6.48%, respectively. The EVaR- PC-based strategies’ annualized returns, especially of the radius_0.1_PC, are also significantly higher than that of the two benchmarks Mean and MV, which indicates that our proposed method has important significance in terms of returns. At the same time, from the point of view of risk control, our proposed method also has enough advantages. Although the Std of our proposed strategies are respectively equal to their corresponding EVaR-based strategy and slightly higher than MV’s, our Maximum Drawdown 23.31% and 21.54% are the lowest among all methods. As we all know, due to some exogenous factors, the global financial market indexes almost all experienced significant declines in the first quarter of 2020. In the face of this systemic risk, radius_0.1 and radius_0.5, like other benchmarks, inevitably saw their returns fall sharply. However, we can see from Fig 2 that the decline of the net asset value of the radius_0.1_PC and radius_0.5_PC are significantly lower than that of others, which shows that our methods have effective risk control ability. In addition to comparing returns and risks separately, the Sharpe ratio is a financial indicator that balances both returns and risks. And the bigger the Sharpe ratio, the better. When *θ* = 0.1 and 0.5, our strategies can increase the Sharpe ratio from 0.52 and 0.36 to 0.79 and 0.40, respectively.

**Fig 3:**
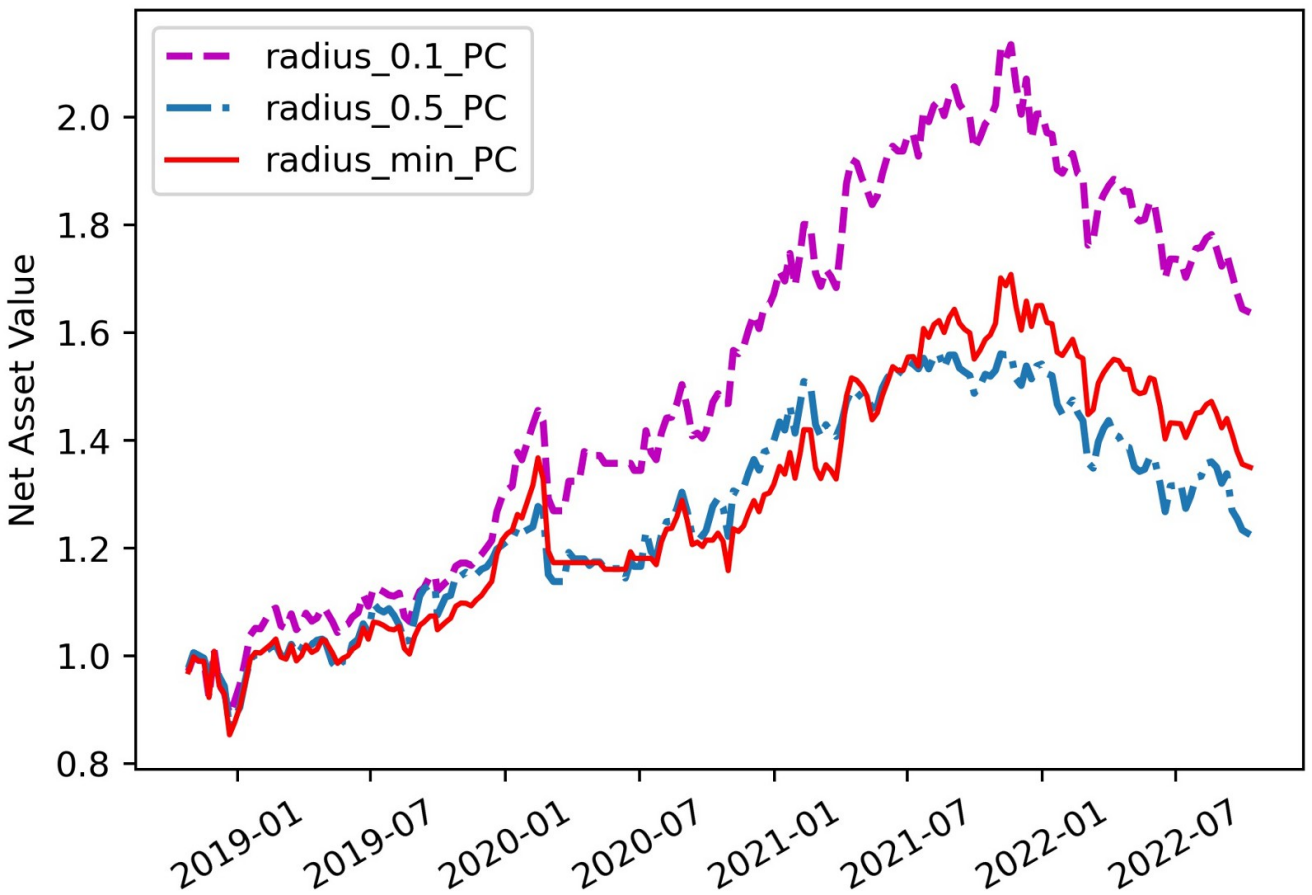
Comparisons of the portfolio performance under different Wasserstein radius.

For the second question, we illustrate it in terms of hyperparameters *θ* and *α*. The *θ* is the radius of ambiguity sets using m-Wasserstein metric and controls the size of the ambiguity sets. In theory, the larger the ambiguity sets, the more conservative the optimal solution is ; but the smaller it is, the lower the probability that the ambiguity sets contain the true distribution. Here, we compare the performance of the portfolio under different Wasserstein radius (i.e., *θ* = 0.1, 0.5, *θ*_*N*_(*β*)). The strategies based on *θ* = *θ*_*N*_(*β*) are called radius_min and radius_min_PC. It can be seen from the Table 1 that, consistent with the conclusions of *θ* = 0.1 and 0.5, the radius_min_PC is superior to the strategy radius_min. Comparing the two strategies, not only did the annualized return increase from 9.21% to 9.23%, but the Maximum Drawdown decreased by about 8.61% from 29.58% to 20.97%. In terms of these EVaR- PC-based strategies, Fig 3 shows that the net asset value curves of radius_0.1_PC and radius_min_PC are higher than that of radius_0.5_PC, which means that the first two strategies are less conservative than the latter. Meanwhile, we can observe from the Table 1 that all strategies with Wasserstein ambiguity set have significantly higher Sharpe ratios than the two benchmarks including Mean(0.31) and MV(0.09). This also suggests that the Wasserstein radius we have chosen are large enough for the Wasserstein ball to contain the true distribution and result in good performance.

**Fig 4:**
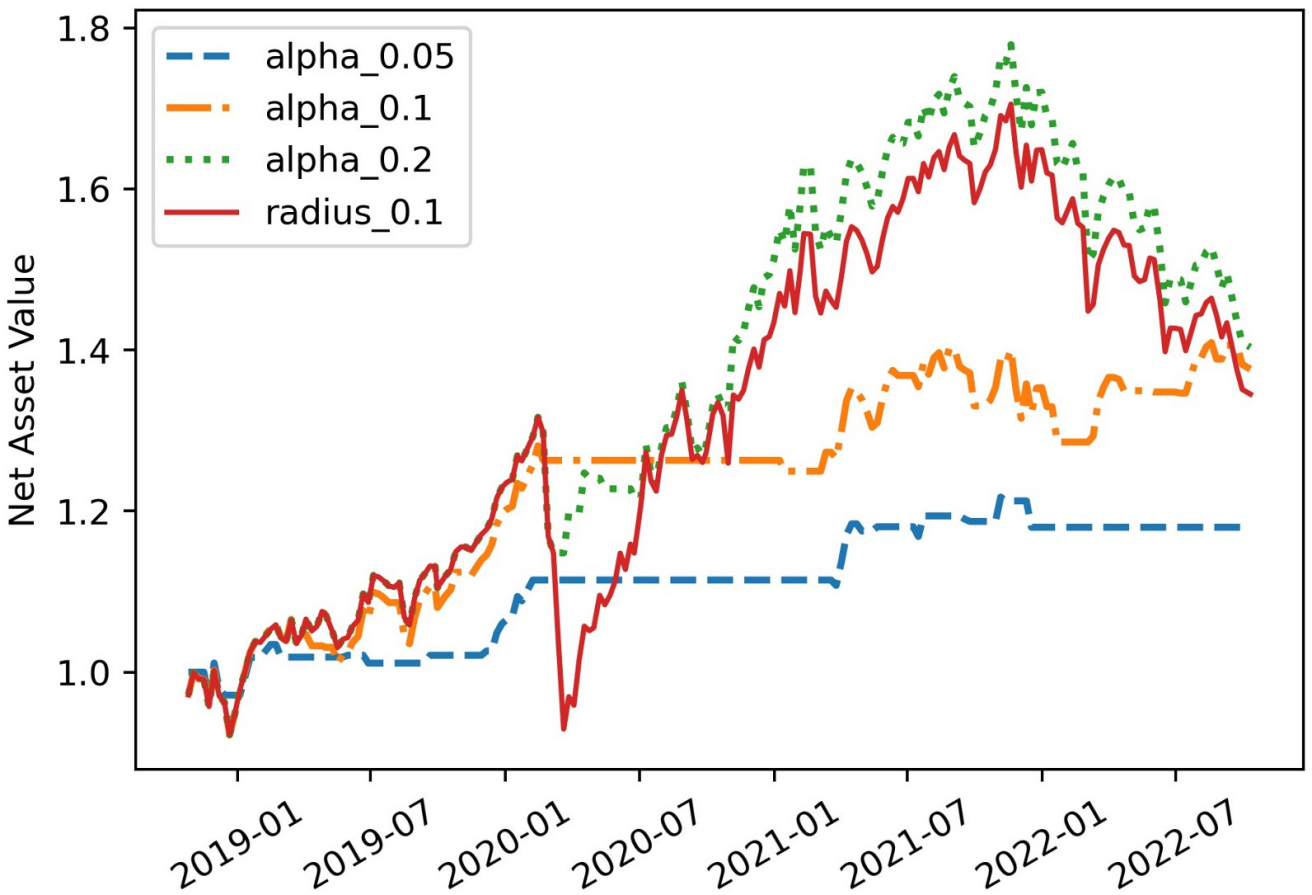
Comparisons of different net asset value curves for different *α*. Let *α* = 0.05, 0.1 and 0.2, and *θ* = 0.1. For alpha_0.05, ***x*** = x* if $\sum_{j=1}^{N}p*(\xi_{j})\Phi_{\text{EVaR-PC}}(\xi_{j}^{⊤}x*,t)\leq 0.05$, otherwise ***x*** = **0**. For alpha_0.1, ***x*** = x* if $\sum_{j=1}^{N}p*(\xi_{j})\Phi_{\text{EVaR-PC}}(\xi_{j}^{⊤}x*,t)\leq 0.1$, otherwise ***x*** = **0**. For alpha_0.2, ***x*** = x* if $\sum_{j=1}^{N}p*(\xi_{j})\Phi_{\text{EVaR-PC}}(\xi_{j}^{⊤}x*,t)\leq 0.2$, otherwise ***x*** = **0**.

To clearly observe the influence of *α* on the experimental results, we present the net asset value curves of strategies with different *α* in Fig 4 and show their corresponding measures in Table 2.

**Table 2:** The performance of methods with different *α* (Annualization).

	*Returns*	*Std*	*Sharpe*_*ratio*	*MaximumDrawdown*
radius_0.1	9.15%	0.18	0.52	29.42%
alpha_0.05	4.37%	0.05	0.80	3.95%
alpha_0.1	8.63%	0.10	0.88	8.28%
alpha_0.2	9.80%	0.15	0.65	21.13%

In this part, we take radius_0.1 as the benchmark. Theoretically speaking, the smaller *α* is, the stricter the risk control is. In the Fig 4, we can find that when *α* is larger, the net asset value curve is higher and its fluctuation is larger. Specifically, the annualized return 9.15% of radius_0.1 is less than that of alpha_0.2’s 9.8% and more than that of alpha_0.05’s 4.37% and alpha_0.1’s 8.63%. In terms of risk, the Std and Maximum Drawdown of alpha_0.05 and alpha_0.1 are dramatically lower than those of alpha_0.2. In particular, the Std and Maximum Drawdown of alpha_0.05 are only 0.05 and 3.95%, respectively. In general, these experimental conclusions are basically consistent with the theoretical properties of *α*. In addition, the net asset value curves of alpha_0.05 and alpha_0.1 didn’t go down at all during the systemic risk in the first quarter of 2020, while the net asset value curve of alpha_0.2 shows obvious fluctuations. And for Sharpe ratio, the alpha_0.2 is also significantly lower among the three methods with different *α*. Therefore, the alpha_0.2 may be too risky for a risk-averse investor.

In general, our EVaR- PC approximation can more accurately describe the violation probability and increase the risk control capability. Compared with the original EVaR-based investment weights, the main difference of our algorithm strategy is that: When the violation probability is less than 5%, we increase the investment weights; when the violation probability is greater than 20%, we give up investment to avoid risks. For investors with different levels of risk aversion, different hyperparameters related to decision making may be selected, such as risk thresholds.

## Conclusions

We investigate the robust chance constrained optimization problem, which plays an important role in modeling uncertain parameters within a decision-making framework. The chance constraint can tell us the violation probability of the event, so as to help us make the optimal decision and avoid the risk. However, since the chance constraint is difficult to evaluate, many conservative approximations have been proposed, such as EVaR and CVaR. To more accurately describe the risk, we propose the PC approximation, which can enjoy nice properties of the DC approximation but are more flexible to introduce more variants. In this paper, we evaluate the EVaR- PC approximation through the discrepancy-based ambiguity sets using m-Wasserstein metric. The discrepancy-based ambiguity set can make full use of the data to estimate the nominal distribution and reduce the sensitivity of decisions to priori knowledges. And we show how to apply our RCCOP model in portfolio management. The numerical experiment results show that our EVaR- PC approximation can describe the violation probability more accurately and help us improve the risk control ability. Investors with different risk preferences can choose different risk thresholds to adjust the style of their strategies.

Theoretically, our proposed EVaR- PC effectively reduces the conservatism of EVaR. In addition, we use m-Wasserstein metric-based ambiguity sets that help improve the robustness of decisions. In practice, the experimental algorithms that we show can provide necessary help for portfolio management. We can continue our research in two aspects in the future: (1) Integrating with data mining and machine learning to full use of the value of data. (2) Thinking about how to make the ambiguity sets more suitable for the case of small samples.
